# MicroRNA-98-5p modulates cervical cancer progression via controlling PI3K/AKT pathway

**DOI:** 10.1080/21655979.2021.2000722

**Published:** 2021-12-11

**Authors:** RongXin Xiao, Hong Wang, Biao Yang

**Affiliations:** Department of Gynaecology, Funing People’s Hospital, Yancheng City, JiangSu Province, China

**Keywords:** MicroRNA-98-5p, PI3K/Akt, cervical cancer, cell proliferation

## Abstract

To probe into the potential mechanism of microRNA (miR)-98-5p inhibiting the biological progress of cervical cancer (CC) cells via regulating PI3K/Akt pathway. Reverse transcription quantitative polymerase chain reaction was applied to detect miR-98-5p expression in CC tissues and cell lines; Cell counting kit-8 and Edu analysis were performed for checking cell proliferation, flow cytometry for cell apoptosis, transwell for cell invasion and migration, Western blot for proliferation-related proteins Ki67 and Proliferating cell nuclear antigen expression, apoptosis-related proteins Bcl-2 and Bax expression, epithelial–mesenchymal transition (EMT)-related proteins Snail, matrix metalloproteinase-3, E-cadherin and N-cadherin expression, as well as PI3K/Akt pathway-related proteins PTEN, PI3K as well as Akt expression levels, and the nude mouse tumor xenograft experiment was applied to verify *in vivo*. The result clarified, miR-98-5p was reduced in CC. Overexpression miR-98-5p could inhibit CC cell proliferation, invasion, migration and EMT, whereas promoted its apoptosis, but silencing miR-98-5p was opposite. Overexpression miR-98-5p could depress the activation of PI3K/Akt pathway in CC *in vivo* and *in vitro*. MiR-98-5p targeted CBX5. In short, miR-98-5p is able to be used as a potential target for treating CC in future research.

## 1. Introduction

Cervical cancer (CC), a malignant gynecological tumor, accounts for a large proportion of female deaths [1–3]. There are many reasons for CC, such as human papillomavirus (HPV) infection, multiple sexual partners, tobacco, etc [4]. Currently, HPV vaccination is the most effective way to prevent and control CC [5]. Although vaccination is available to effectively control CC incidence, vaccination is a serious economic burden for those in low-income areas and countries [6,7]. Therefore, finding new therapeutic targets and exploring their mechanism may be beneficial for treating CC.

MicroRNA (miRNA), a non-coding RNA of 20–24 nt in length, affects the course of disease through influencing the post-transcriptional translation of downstream mRNA [8,9]. Based on large number of works, various miRNAs are differentially expressed in CC and exhibit their ability to regulate multiple biological functions of CC. For example, Yu F et al. found that reduced miR-145 and elevated miR-205 are related to CC malignancy infected by HPV [10]. Recently, it was found that miR-519d-3p is available to elevate CC sensitivity to cisplatin [11]. Additionally, miR-338-3p [12], miR-663b [13], miR-769-5p [14], miR-133b [15], miR-101 [16], miR195-5p [17] and other miRNAs have been found to be related to CC progression. MiR-98-5p is an essential miRNA. Based on a recent research, miR-98-5p interacts with 22 circular RNA (circRNA) transcripts in CC tissues, indicating that the biological functions of miR-98-5p may be abundant in CC progression [18]. But the specific biological function of miR-98-5p in CC is still unclear.

In the research, the aim was to probe into miR-98-5p’s potential mechanism in controlling CC progression. For this end, miR-98-5p was changed in CC cell lines via transfection. In addition, there were examined changes in cancer cell proliferation, invasion, migration and EMT, with focus on whether miR-98-5p affects PI3K/AKT pathway in CC.

## 2. Methods

### 2.1. Tissue samples & ethical approval

Total 58 cases of CC tissues as well as normal adjacent tissues were gained from patients undergoing surgery in Funing People’s Hospital. After the resection, CC specimens and the matched normal tissue specimens adjacent to the cancer were quickly frozen in liquid nitrogen, and the clinical data of patients are represented in Table 1. Cervical tumor tissues were taken from patients with primary cervical tumor, and all patients were diagnosed by at least two experienced pathologists. This research has got Ethics board approval of Funing People’s Hospital and was constructed in line with the *Declaration of Helsinki*. All patients signed the informed consent.

**Table 1.** Correlation analysis between miR-98-5p and clinicopathological characteristics of CC patients

			MiR-98-5p expression		
Characteristic	Group	Cases	High expression group (n = 29)	Low expression group (n = 29)	*P*
Age	< 50	18	10	8	0.5702
	50 or more	40	19	21	
Tumor size (cm)	< 5 cm	41	23	18	0.1492
	5 cm or more	17	6	11	
Histological grade	Moderate	31	18	13	0.1881
	Low	27	11	16	
TNM staging	I + II	34	27	7	< 0.0001
	III + IV	24	2	22	
Lymph metastasis	Yes	12	2	10	0.0095
	No	46	27	19	

Pearson’s correlation coefficient analysis was applied to investigate the relationship between miR-98-5p and the clinicopathological characteristics of CC patients.

### 2.2. Cell culture

Human normal cervical epithelial cells (HCerEpiC) as well as CC cell lines (CaSki, HT-3, C33A, SiHa) were purchased from Chinese Academy of Sciences, Shanghai Institute of Biochemistry and Cell Biology, and the cells were cultured in Dulbecco’s Modified Eagle Medium (DMEM) = (Cellmax). All media contained 10% fetal bovine serum (FBS), 100 U/mL penicillin and streptomycin (Invitrogen). The cells were confirmed that they did not contain mycoplasma and were incubated with 5% CO_2_.

### 2.3. Cell transfection

CaSki and SiHa cells (1 × 10^5^ cells/well) were cultivated in a 24-well plate. In line with the instructions, miR-98-5p mimic/inhibitor and negative control (NC) (GENEWIZ, China) were transfected into CaSki and SiHa cell via Lipofectamine 2000 (Thermo Fisher Scientific, U.S.). After 48-hour-transfection, the cells were harvested for subsequent experiments.

### 2.4. Reverse transcription quantitative polymerase chain reaction (RT-qPCR)

Performance of RT-qPCR was as set forth [19]. TRIzol reagent (Invitrogen) was applied to extract total RNA from tissues or cells, with PrimeScript™ RT Master Mix (Takara, China; RR036Q) to perform reverse transcription of RNA into cDNA. RT-qPCR was performed through SYBR®Premix exTaq™ (Baokala) under the StepOnePlus™ real-time PCR system (Applied Biosystems, U.S.), with U6 as an internal miRNA control, and the relative quantification method (2^−ΔΔCt^) was conducted to calculate gene expression, and the primer sequences were presented in Table 2.

**Table 2.** RT-qPCR primer sequences

	Primer sequences (5ʹ – 3ʹ)
GAPDH	Forward: 5ʹ-ATGGGGAAGGTGAAGGTCG-3’
	Reverse: 5ʹ-TTACTCCTTGGAGGCCATGTG-3’
U6	Forward: 5ʹ-CTCGCTTCGGCAGCACATATACT-3’
	Reverse: 5ʹ-ACGCTTCACGAATTTGCGTGTC-3’
Bcl-2	Forward: 5ʹ- CTGGTGGACAACATCGCTCTG −3’
	Reverse: 5ʹ- GGTCTGCTGACCTCACTTGTG −3’
MiR-98-5p	Forward: 5ʹ- TGAGGTAGTAGTTTGTGCTGTT-3’
	Reverse: 5ʹ- GCGAGCACAGAATTAATACGAC-3’
Bax	Forward: 5ʹ- GGATCGAGCAGAGAGGATGG −3’
	Reverse: 5ʹ- TGGTGAGTGAGGCAGTGAGG −3’
CBX5	Forward: 5ʹ- GCAGACGTTAGCGTGAGTG −3’
	Reverse: 5ʹ- GCGGAATTCGGATCCCTCGAGTT-3’

### 2.5. Cell counting kit (CCK)-8 assay

Detecting cell proliferation was conducted as described previously [20]. CaSki and SiHa cells were seeded into 96-well plates (3000 cells/well), added with CCK-8 solution (Dojindo) to each well, and incubated for 1 hour at 0, 24, 48, 72 and 96 hours after transfection. A spectrophotometer (Thermo Fisher) was used to measure the optical density (OD) value at 450 nm, and each determination was performed in triplicate and three times were repeated independently.

### 2.6. 5-Ethynyl-2ʹ-deoxyuridine (EdU) proliferation assay

In accordance with the instructions, an EdU analysis kit (Ribobio) was applied to evaluate CaSki and SiHa cell proliferation. The cells were incubated with 50 μM EdU at 37°C, later fixed with 4% formaldehyde, and permeabilized with 0.5% Triton X-100 at room temperature for 20 minutes. The cells were later incubated with the 1 × Apollo reaction mixture. After incubating, the DNA were stained with 4ʹ, 6-diamidino2-phenylindole, and EdU-positive cells were observed under fluorescence microscopes (Carl Zeiss).

### 2.7. Flow cytometry to detect apoptosis

CaSki and SiHa cells were seeded (1 × 10^4^ cells/well) into 96-well plates, and in accordance with the instructions, Annexin V- fluorescein isothiocyanate (FITC) Apoptosis Detection Kit (Sigma-Aldrich; APOAF-50TST) was applied to detect apoptosis. The cells were double stained with Annexin V-FITC and propidium iodide (PI) and apoptotic cells were tested via flow cytometry (Becton-Dickinson, USA).

### 2.8. Transwell experiment

For the invasion assay, the upper chamber of the 24-well Transwell (Corning Life Sciences, USA) was covered with Matrigel, later CaSki and SiHa cells were seeded, and added with fresh 600 µL of DMEM containing 10% FBS to the lower chamber. After 24-hour-incubation, the cells were fixed and stained with methanol as well as crystal violet (Sigma-Aldrich), and the invading cells from the chambers were counted under inverted microscopes (Olympus). In the migration assay, the 24-well Transwell chamber was not covered by Matrigel, and the remaining steps were the same as those in the invasion assay.

### 2.9. Western blot

The total protein was lysed via Radio-Immunoprecipitation assay lysis buffer (Sigma-Aldrich), separated with sodium dodecyl sulfate polyacrylamide gel electrophoresis, and transferred to a Polyvinylidene fluoride (PVDF) membrane (Abcam, UK). The membrane was immersed in 5% skim milk (Thermo) for 3 h to block nonspecific protein binding, combined with the following antibodies: Phospho-PI3K (p-PI3K, 4228, Cell Signaling Technology), PI3K (4257, Cell Signaling Technology), p-Akt (4060, Cell Signaling Technology), Akt (9272, Cell Signaling Technology), glyceraldehyde-3-phosphate dehydrogenase (GAPDH) (60,004-1-Ig, Proteintech), ki-67 (b15580, Abcam), Proliferating cell nuclear antigen (PCNA) (ab29, Abcam), N-cadherin (ab18203, Abcam), E-cadherin (ab1416, Abcam), Bcl-2 (sc-7382, Santa Cruz Biotechnology), Bax (ab32503, Abcam), PTEN (9559, Cell Signaling Technology), Snail (3879, Cell Signaling Technology), matrix metalloproteinase-3 (MMP-3) (ab52915, Abcam), and incubated overnight. The PVDF membrane was incubated with the secondary antibody (Abcam, ab205718). Enhanced chemiluminescence reagent (Sigma-Aldrich) was applied to detect protein bands, and protein expression was analyzed under Image J software (NIH, USA).

### 2.10. Dual luciferase reporter assay

The assay was conducted as described previously [21]. The luciferase reporter vector consisting of full length CBX5 3ʹ-untranslated region (3ʹ-UTR) sequence was obtained from RiboBio (Guangzhou, China). QuikChange site-directed mutagenesis kit (Stratagene, CA, USA) was applied to generate mutant luciferase reporter vector. According to the manufacturer’s method, the above-mentioned reporter vector and miR-98-5p mimic/NC were co-transfected into CaSki cells using Lipofectamine 2000 (Thermo Fisher Scientific, US). Following 48 h transfection, the Firefly and Renilla luciferase activities were measured using the Dual-Luciferase Reporter Assay System (Promega).

### 2.11. RNA immunoprecipitation (RIP) assay

EZ-Magna RIP kit (Millipore, USA) was applied for RIP determination [22]. In short, the cells were lysed in RIP lysis buffer and then incubated in RIP buffer containing magnetic beads coupled to a negative control (normal mouse Immunoglobulin G (IgG)) or human anti-Argonaute 2 (Ago2, Millipore). Subsequently, the co-precipitated RNA was separated and purified, and finally the RNA enrichment was detected by RT-qPCR.

### 2.12. Tumor xenograft experiment in nude mice

Animal experiments were implemented based on the Guide for the Care and Use of Laboratory Animals, with approval from the Animal Care and Use Committee of Funing People’s Hospital. Four-week-old female BALB/c nude mice (Shanghai National Laboratory Animal Center, China) were raised in sterile environment at 24 ± 2°C, with 50%-60% humidity, and alternating light and shade for 12 hours. The Caski (5 × 10^6^) transfecting miR-98-5p mimic or inhibitor were injected subcutaneously into the right side of each mouse, and the tumor volume was measured every week, and the tumor volume was counted via the following formula: Volume (mm^3^) = (length × width)^2^/2. After 6 weeks, the animals were euthanized via cervical dislocation and the tumor were placed in formalin for fixation (Figure S1). In order to observe the distant metastasis of the tumor, CaSki cells (3 × 10^5^) transfected with miR-98-5p mimic or inhibitor were injected into nude mice via tail vein. After 8 weeks, the lung tissue was removed, embedded in conventional paraffin and Hematoxylin-eosin (HE) staining was conducted to observe the location of tumor metastasis in the lung [23].

### 2.13. Immunohistochemistry

The tumor tissue was embedded in paraffin, and the paraffin-embedded sections were degreased, blocked with xylene, and later dehydrated via a graded alcohol series. The tissue sections were incubated with 10% goat serum, later the following primary antibodies overnight at 4°C: P-PI3K (4228, Cell Signaling Technology), p-Akt (4060, Cell Signaling Technology) goat anti-rabbit secondary antibody conjugated with horseradish peroxidase. The sections were added with 3-diaminobenzidine tetrahydrochloride and counterstained with hematoxylin. Positive cells appeared yellow or brown.

### 2.14. Data analysis

The experimental results were presented as mean ± standard deviation (SD), SPSS 22 software was applied for data analysis, including Student’s t test and one-way analysis of variance (ANOVA), as well as Tukey’s test to correct variance for multiple times on samples. Pearson’s correlation coefficient analysis was applied for investigating the relationship between miR-98-5p and the clinicopathological characteristics of CC patients. The difference between the experimental groups was considered significant when *P* < 0.05.

## 3. Results

### 3.1. Reduced miR-98-5p is in CC

**Figure 1.**
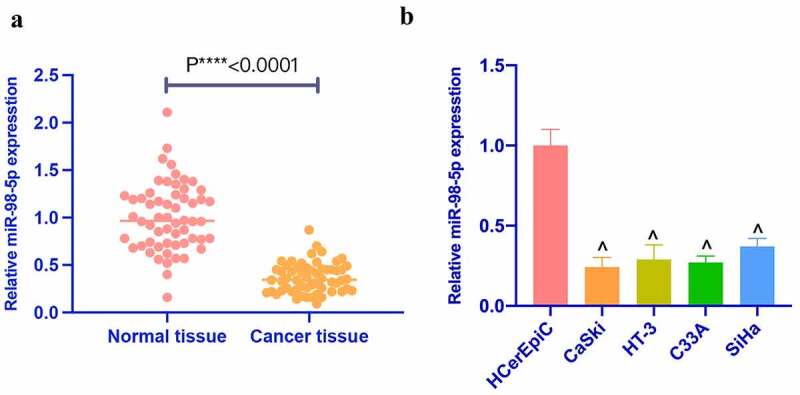
Reduced miR-98-5p is present in CC. (a) RT-qPCR to detect miR-98-5p expression in CC tissues as well as adjacent normal tissues; (b) RT-qPCR to detect miR-98-5p expression levels in HCerEpiC and CC cell lines, CaSki, HT-3, C33A, and SiHa. The values presented as mean ± SD (n = 3). One-way ANOVA was applied for calculating the significance of each group, with Tukey’s test for corrected variance. Vs. normal tissue group, *P < 0.05; Vs. HcerEpiC group ^*P* < 0.05

MiR-98-5p was first checked and reduced in CC tissues. Additionally, miR-98-5p was also detected in CC cell lines (Figure 1a). In addition, based on the median of miR-98-5p expression, 58 CC patients were divided into miR-98-5p high expression and low expression groups to analyze whether the abundance of miR-98-5p was related to tumor prognosis. Table 1 clarified that miR-98-5p was related to TNM staging and metastatic lymph node ratio, but had nothing to do with other clinicopathological characteristics, such as age, tumor size, and histological grade. MiR-98-5p in four CC cell lines (CaSki, HT-3, C33A, SiHa) was lower than that in HCerEpiC (Figure 1b). Among four CC cell lines, miR-98-5p was expressed the lowest in CaSki and the highest in SiHa among. Therefore, CaSki and SiHa cells were selected for subsequent functional tests.

### 3.2. MiR-98-5p represses CC proliferation

**Figure 2.**
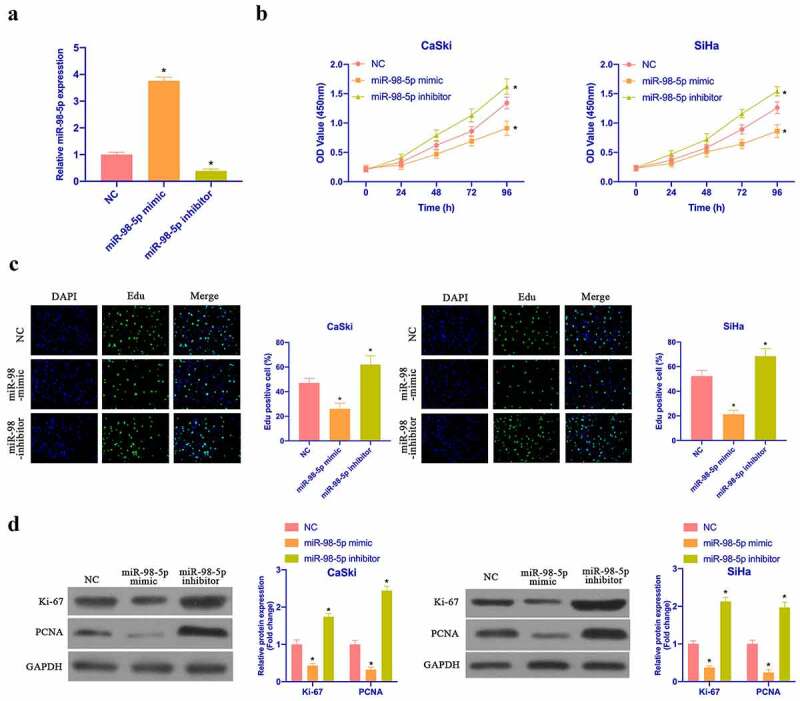
MiR-98-5p inhibits CC proliferation. (a) RT-qPCR to detect miR-98-5p expression in CaSki and SiHa cells in the NC, miR-98-5p mimic and miR-98-5p inhibitor groups; (b) CCK-8 to detect CaSki and SiHa cell proliferation in the NC, miR-98-5p mimic and miR-98-5p inhibitor groups; (c) Colony formation test to detect the number of CaSki and SiHa cell colonies in the NC, miR-98-5p mimic and miR-98-5p inhibitor groups; (d) Edu analysis to detect the of CaSki and SiHa cell proliferation in the NC, miR-98-5p mimic and miR-98-5p inhibitor groups; (e) Western blot to detect ki-67 and PCNA protein expression in CaSki and SiHa cells in the NC, miR-98-5p mimic group and miR-98-5p inhibitor groups. The values presented as mean ± SD (n = 3). One-way ANOVA was applied to calculate the significance of each group, with Tukey’s test for corrected variance. Vs. NC group, *P < 0.05

Aiming to examine the impact of miR-98-5p on CC proliferation, miR-98-5p mimic and miR-98-5p inhibitor were transfected to up- as well as down-regulate miR-98-5p in CaSki and SiHa cells, respectively (Figure 2a). Subsequently, CCK-8, colony formation test and Edu test were applied to examine the impact of overexpressing or silencing miR-98-5p on CaSki and SiHa cell proliferation. As shown in Figure 2b–d), the proliferation rate and the cell colonies of CaSki and SiHa cells, with the number of Edu-positive cells were elevated via overexpressing miR-98-5p, while silencing miR-98-5p had the opposite impact. CaSki and SiHa cell proliferation-related protein expression was checked via Western blot. Overexpressing miR-98-5p inhibited ki-67 and PCNA protein content in CaSki and SiHa cells, while silencing miR-98-5p elevated ki-67 and PCNA protein content (Figure 2e). This implies that miR-98-5p can inhibit CC proliferation.

### 3.3. MiR-98-5p promotes CC apoptosis

**Figure 3.**
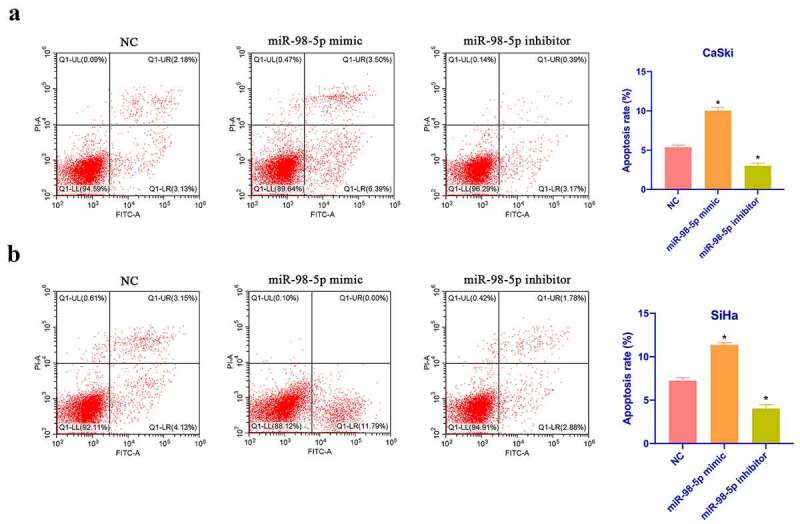
MiR-98-5p promotes CC apoptosis. (a & b): Flow cytometry to check CaSki and SiHa cell apoptosis rate in the NC, miR-98-5p mimic and miR-98-5p inhibitor groups; (c & d): RT-qPCR as well as Western blot to detect Bcl-2 and Bax expression levels in CaSki and SiHa cells in the NC, miR-98-5p mimic and miR-98-5p inhibitor groups. The values presented as mean ± SD (n = 3). One-way ANOVA was applied to calculate the significance of each group, with Tukey’s test for corrected variance. Vs. NC group,*P < 0.05

Next, CaSki and SiHa cell apoptosis was checked via flow cytometry. As shown in Figure 3a and b, overexpressing miR-98-5p promoted CaSki and SiHa cell apoptosis, whereas silencing miR-98-5p inhibited that. Additionally, based on Western blot findings, overexpressing miR-98-5p elevated Bax expression in CaSki and SiHa cells but decreased Bcl-2 expression, whereas silencing miR-98-5p had the opposite impact (Figure 3c & d). This implies that miR-98-5p is available to promote CaSki and SiHa cell apoptosis.

### 3.4. MiR-98-5p refrains CC invasion, migration and EMT

**Figure 4.**
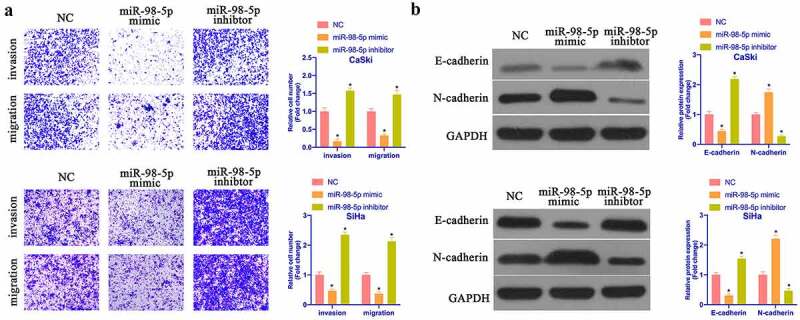
MiR-98-5p inhibits CC invasion, migration and EMT. (a) Transwell to detect CaSki and SiHa cell invasion and migration in the NC, miR-98-5p mimic and miR-98-5p inhibitor groups; (b) Western blot to detect EMT-related proteins Snail, MMP-3, E-cadherin and N-cadherin expression in CaSki and SiHa cells in the NC, miR-98-5p mimic and miR- 98-5p inhibitor groups. The values presented as mean ± SD (n = 3). One-way ANOVA was applied to calculate the significance of each group, with Tukey’s test for corrected variance. Vs. NC group, *P < 0.05

Subsequently, the impact of miR-98-5p on CaSki & SiHa cell progression was further examined via Transwell and Western blot. As shown in Figure 4a, overexpressing miR-98-5p promoted CaSki & SiHa cell invasion and migration, while silencing miR-98-5p reduced them. In accordance with Western blot findings, overexpressing miR-98-5p reduced N-cadherin, Snail, and MMP-3 protein but promoted E-cadherin protein, while silencing miR-98-5p had the opposite impact (Figure 4b). It implies that miR-98-5p is available to inhibit CC invasion, migration and EMT.

### 3.5. MiR-98-5p represses PI3K/Akt pathway’s activation in CC

**Figure 5.**
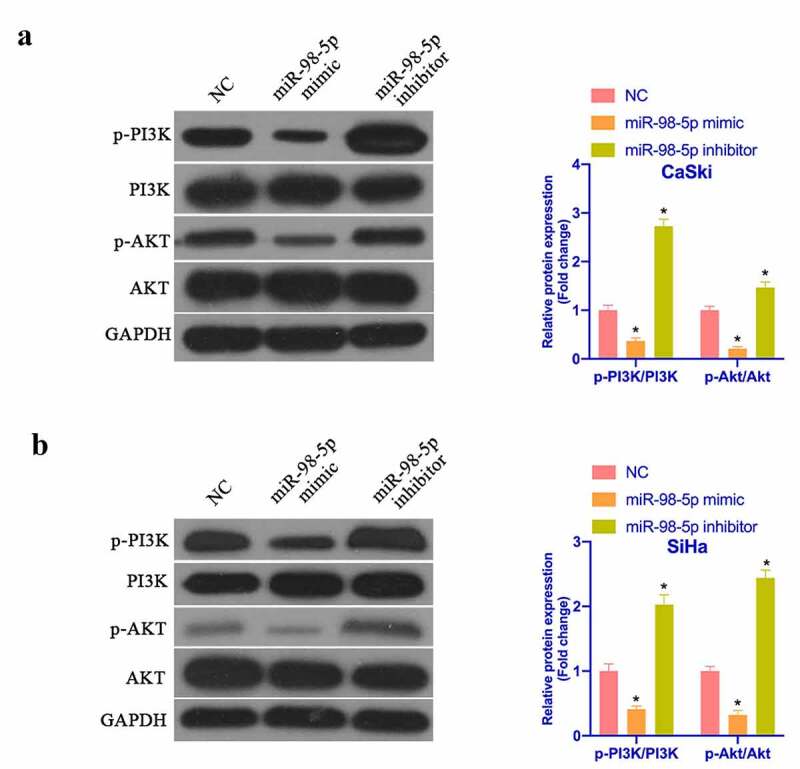
MiR-98-5p inhibits the activation of PI3K/Akt pathway in CC. (a & b) Western blot to detect PTEN, PI3K and Akt protein expression in CaSki and SiHa cells in the NC, miR-98-5p mimic and miR-98-5p inhibitor groups. The values presented as mean ± SD (n = 3). One-way ANOVA was applied to calculate the significance of each group, with Tukey’s test for corrected variance. Vs. NC group, *P < 0.05

It is known that activating PI3K/Akt pathway manifests an essential relationship with the biological progress of CC. PTEN is a recognized tumor suppressor and a vital antagonist of PI3K. Next, the impact of miR-98-5p on PI3K/Akt pathway protein expression was examined via Western blot. As shown in Figure 5a and b, after overexpressing miR-98-5p, p-PI3K and p-Akt protein expression in CaSki and SiHa cells was visually reduced, while PTEN protein expression was visually elevated. Silencing miR-98-5p presented the opposite outcome. This implies that miR-98-5p can depress the activation of the PI3K/Akt pathway in CC.

### 3.6. MiR-98-5p targets CBX5

**Figure 6.**
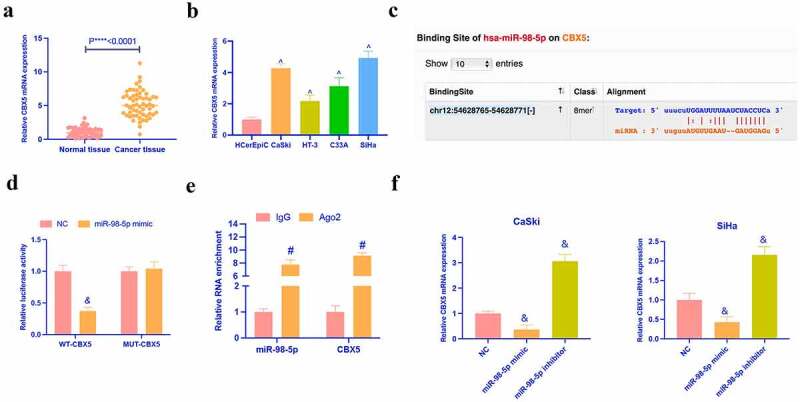
MiR-98-5p targets CBX5. (a, b) RT-qPCR to detect the expression of CBX5 in CC tissues and cells; (c) Potential binding sites of miR-98-5p and CBX5 forecast via bioinformatics website http://starbase.sysu.edu.cn/; (d) Dual luciferase reporter assay to verify the targeting relationship between miR-98-5p and CBX5; (e) RIP experiment to verify the binding relationship between miR-98-5p and CBX5; (f) RT-qPCR detection of the effect of knockdown or overexpression of miR-98-5p on the expression of CBX5 in CC cell line; The values presented as mean ± SD (n = 3). One-way ANOVA was applied to calculate the significance of each group, with Tukey’s test for corrected variance. Vs. normal tissue group, *P < 0.05; Vs. HcerEpiC group, ^*P* < 0.05; Vs. NC group, ^&^*P* < 0.05; Vs. IgG group, ^#^*P* < 0.05

Next, it was explored the target genes of miR-98-5p. CBX5 has been found to aberrantly express in renal cell carcinoma [24], breast cancer [20], and acts as an oncogene. However, its role in CC is unclear. In this study, it was discovered that CBX5 was up-regulated in CC tissues and cells (Figure 6a, b). Meanwhile, Via the prediction of bioinformatics website http://starbase.sysu.edu.cn/, it was discovered that miR-98-5p and CBX5 had latent binding sites (Figure 6c). Dual luciferase reporter assay indicated that the co-transfection of wild-type CBX5 and miR-98-5p would reduce the luciferase activity in CC cells, while that of mutant CBX5 and miR-98-5p mimic had no effect on the luciferase activity (Figure 6d). RIP experiment manifested that there were enriched CBX5 and miR-98-5p in Ago2 magnetic beads (Figure 6e). In addition, in CaSki and SiHa with knocking down or overexpressing miR-98-5p, the expression of CBX5 was found to be promoted and inhibited, respectively (Figure 6f). These data clarified that miR-98-3p targeted CBX5 in CC.

### 3.7. MiR-98-5p restrains CC tumor growth

**Figure 7.**
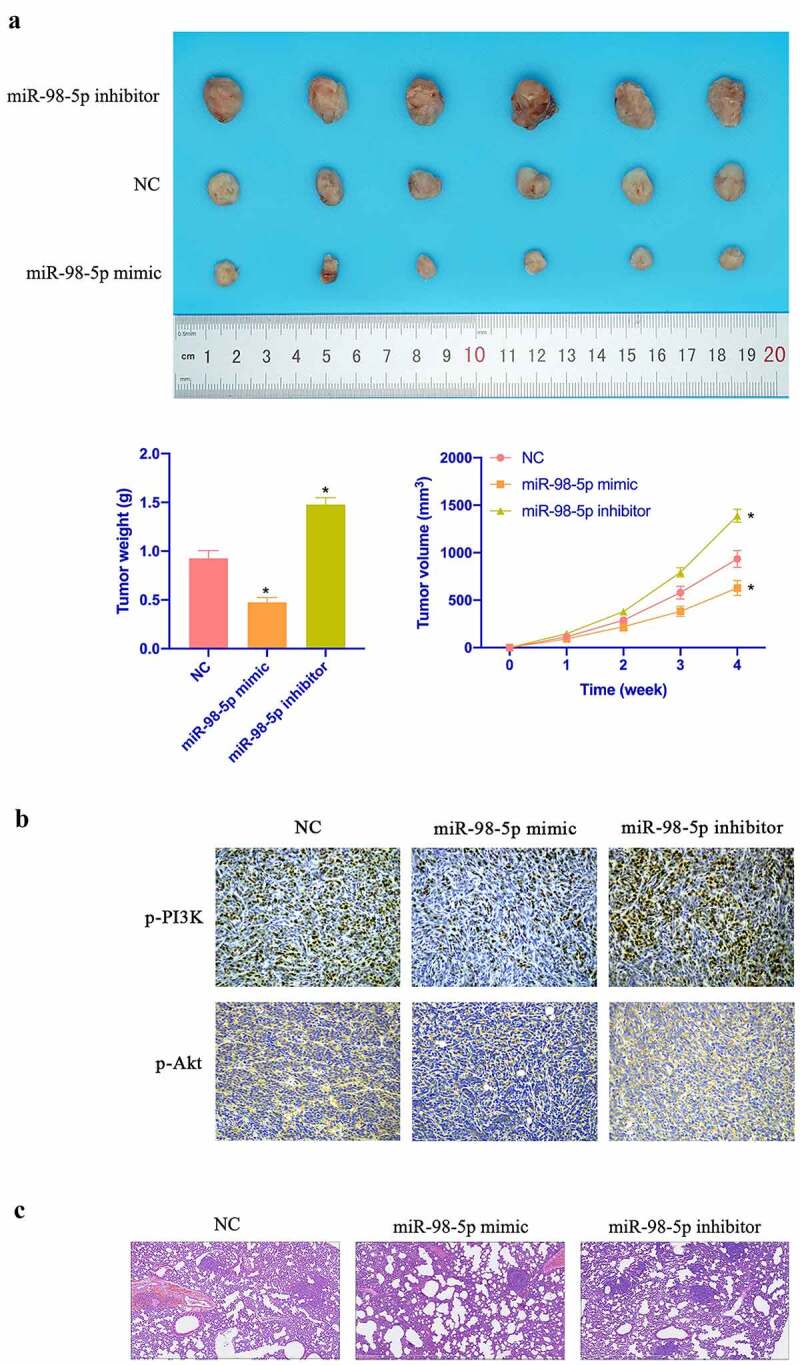
MiR-98-5p inhibits CC growth. (a) Representative image of tumor; (b) Tumor volume; (c) Tumor; (d) Immunohistochemistry to detect PI3K and Akt expression in tumor tissue. Representative pictures of HE staining of lung tissue with distant metastasis of tumor. The values presented as mean ± SD (n = 6). One-way ANOVA was applied to calculate the significance of each group, with Tukey’s test for corrected variance. Vs. NC group,*P < 0.05

Aiming to further verify the *in vitro* findings, *in vivo* experiments were conducted. As shown in Figure 7a–c, overexpressing miR-98-5p inhibited CC growth rate and reduced tumors, while silencing miR-98-5p promoted CC growth. Additionally, overexpressing miR-98-5p also inhibited PI3K and Akt expression in tumor, while silencing miR-98-5p promoted PI3K and Akt expression (Figure 7d & e). In addition, Figure 7f manifested that miR-98-5p knockdown motivated the number of lung metastatic nodules of tumor, while miR-98-5p upregulation reduced the number of lung metastatic nodules. In conclusion, this implies that miR-98-5p can inhibit CC progression *in vivo*.

## 4. Discussion

Preventing and treating CC still faces great challenges. In this research, it was found that miR-98-5p acts as a tumor suppressor in CC, manifesting as repressing CC cell progression through preventing the activation of the PI3K/Akt pathway in CC.

Based on previous researches, miR-98-5p is available to regulate cancer development through various target genes, like XIAP [25], BZW1 [26], STAT3 [27], IGF1 [28] and so on. General speaking, miRNA is available to depress mRNA and protein via binding to the 3ʹUTR end of target genes, thereby affecting cancer progression [29]. In this work, miR-98-5p is available to depress CC proliferation, invasion, migration and EMT. But the downstream target genes modulated by it still need to be further probed into. Additionally, in recent years, it has been found that circRNA can act as a miRNA target gene sponge in the body to regulate its gene expression [30]. Based on a recent study, 11 circRNAs may perform as miR-98-5p’s sponges in CC. There may be more circRNA involved in regulating miR-98-5p expression in CC, which needs to be further explored. Chemoresistance is a serious obstacle in cancer treatment. Many reports have reported that miR-98-5p links with chemoresistance in various cancers, like gastric cancer [31], liver cancer [32], as well as glioma [33]. Therefore, it is necessary to probe into whether miR-98-5p is available to regulate chemoresistance in CC in the future.

Regulating PI3K/Akt signaling pathway is very vital to cell growth, survival, death and metabolism, and the imbalance of this pathway closely links with cancer occurrence and development [34]. Therefore, exploring the targeted modulator of PI3K/Akt pathway has great potential. A great number of PI3K/Akt modulator drug candidates are under development, and some have been applied for treating cancer patients. But PI3K/Akt is still resistant to treatment. In accordance with recent studies, miR-98-5p is available to modulate PI3K/Akt pathway to render hepatocellular carcinoma cells sorafenib-resistant [33]. Additionally, researches have revealed that miR-98-5p is available to improve mesenchymal stem cell apoptosis in immune thrombocytopenia through regulating the PI3K/Akt pathway [35]. In this study, it was found that miR-98-5p is available to suppress the PI3K/Akt pathway’s activation in CC cell lines, thereby intervening in CC cell progression.

## 5. Conclusion

To sum up, the findings revealed that miR-98-5p is available to repress CC cell progression through PI3K/Akt pathway, thereby providing new insights for understanding CC pathogenesis and its future treatment.

## Supplementary Material

Supplemental Material
